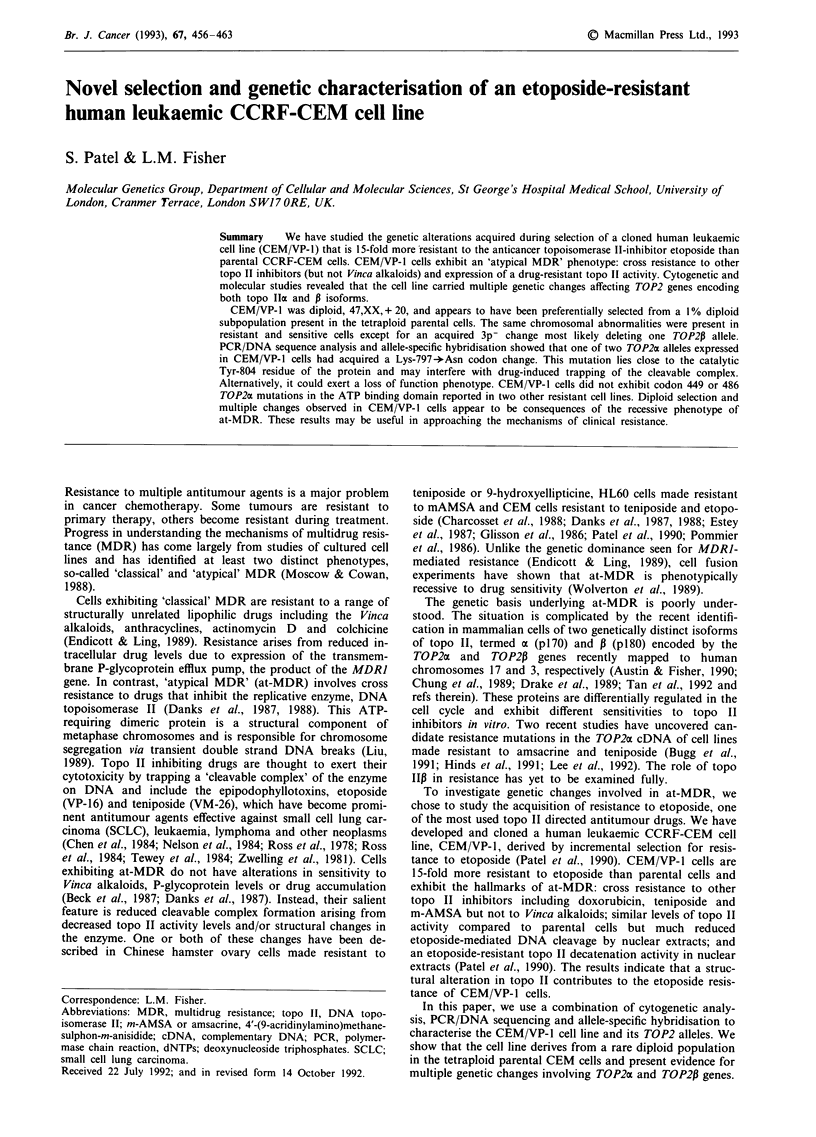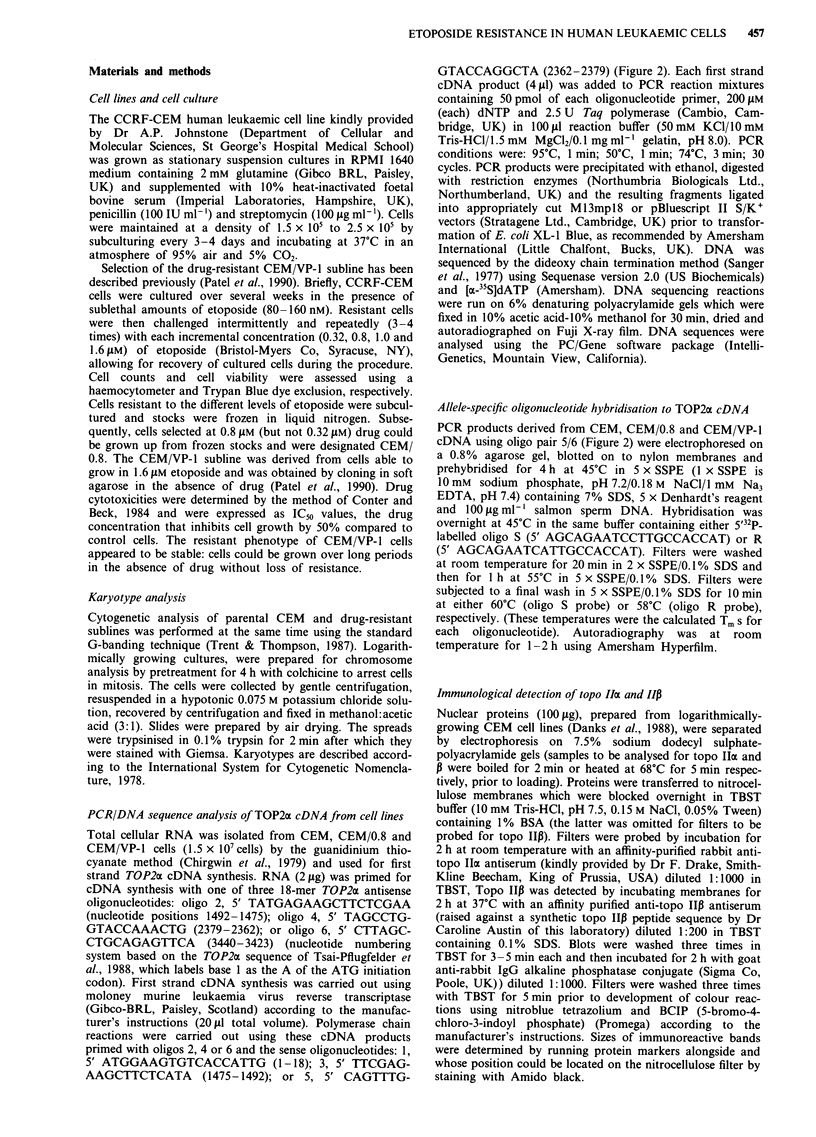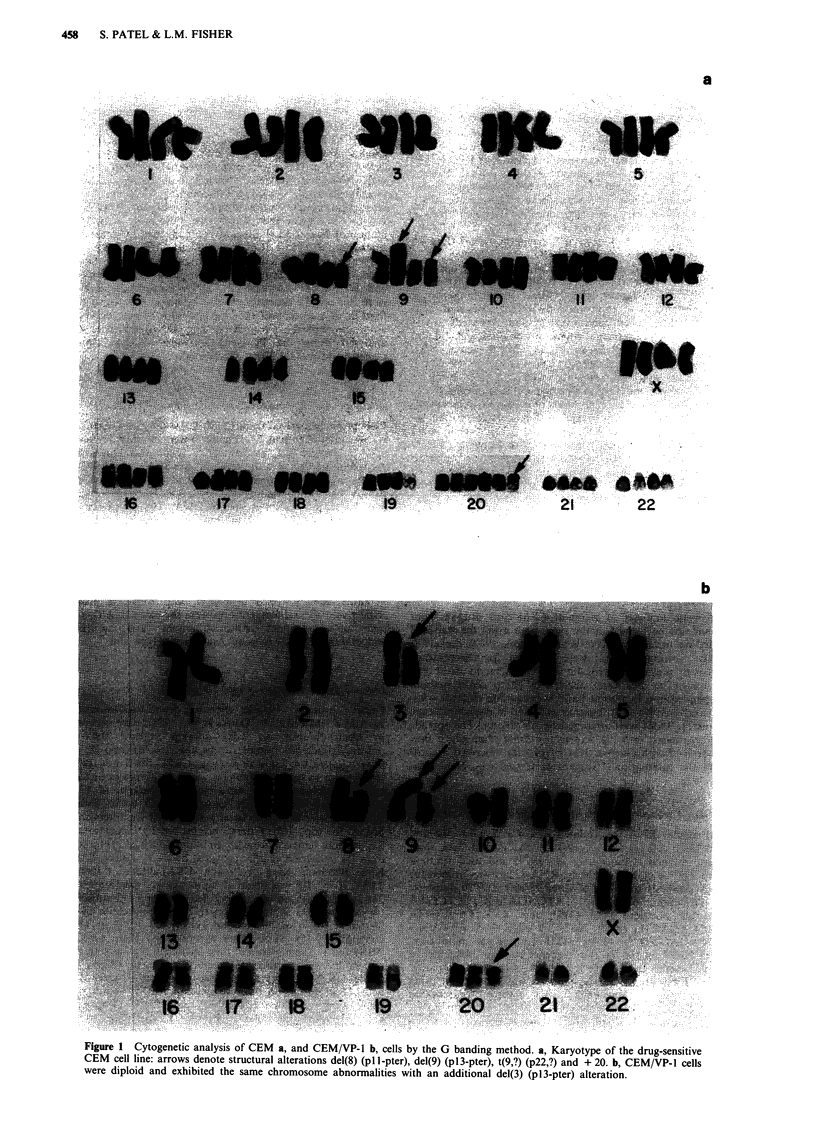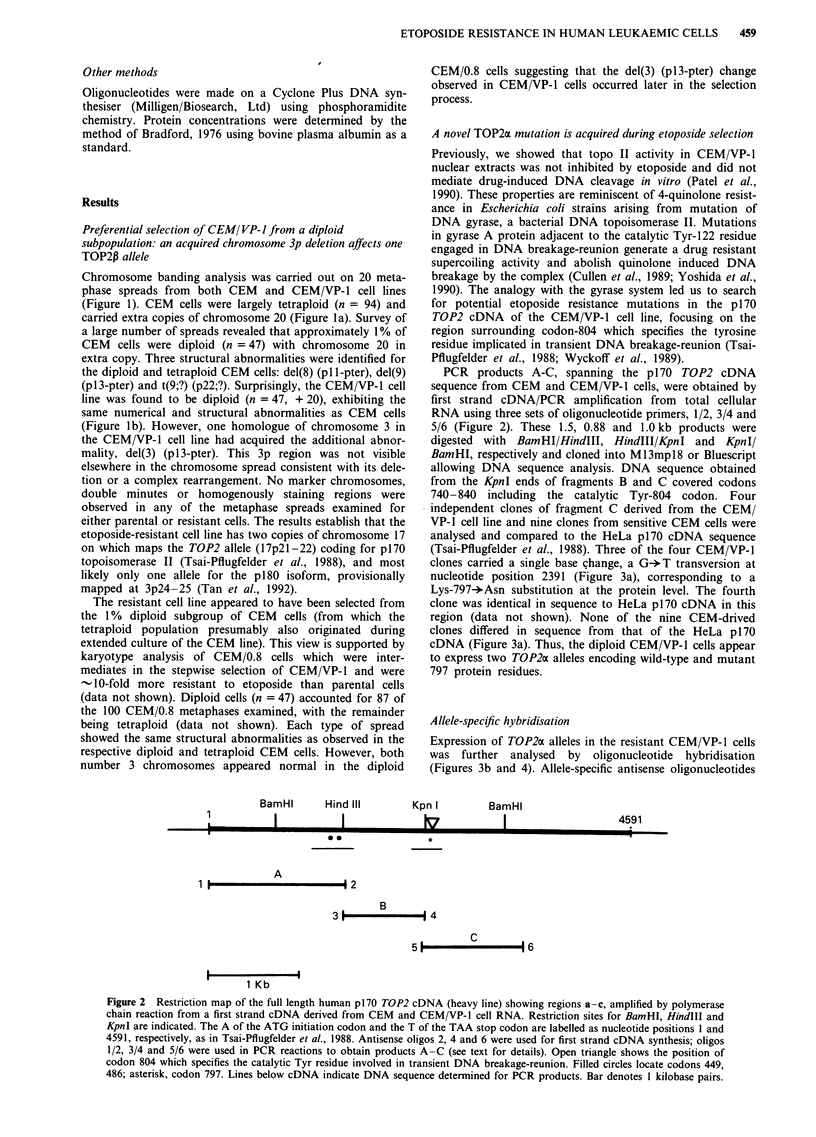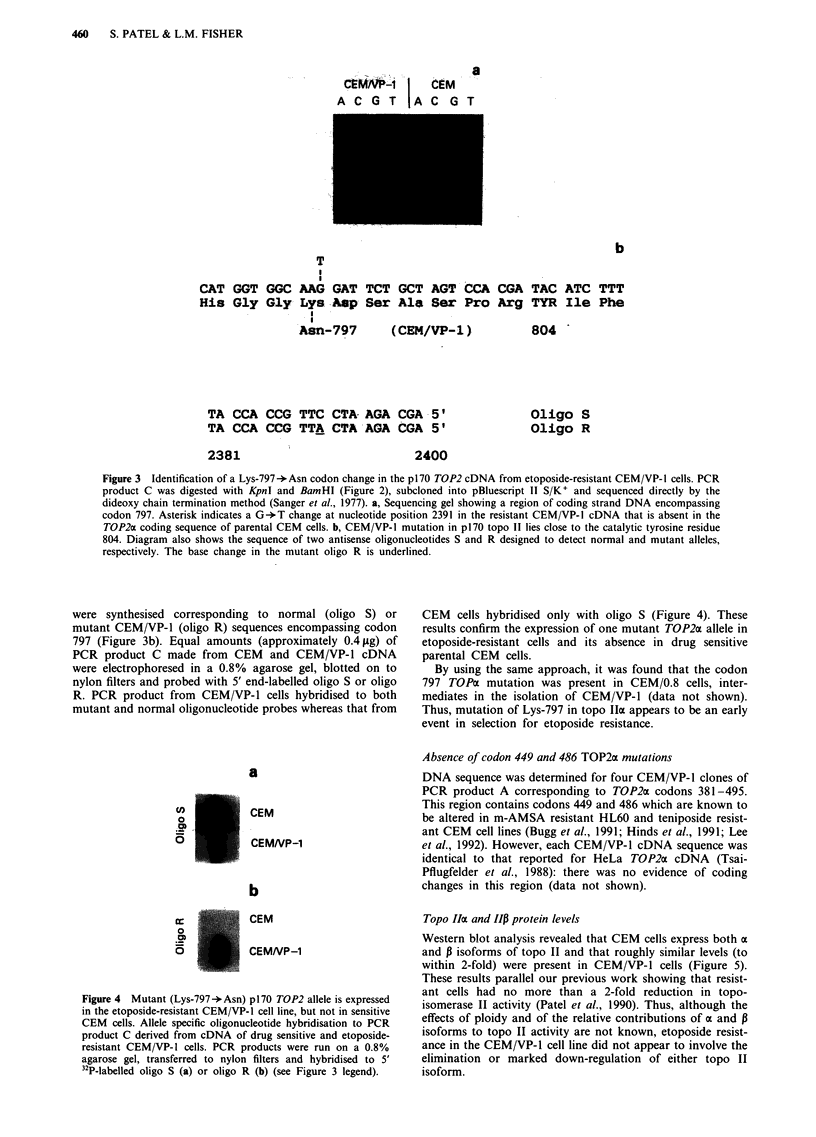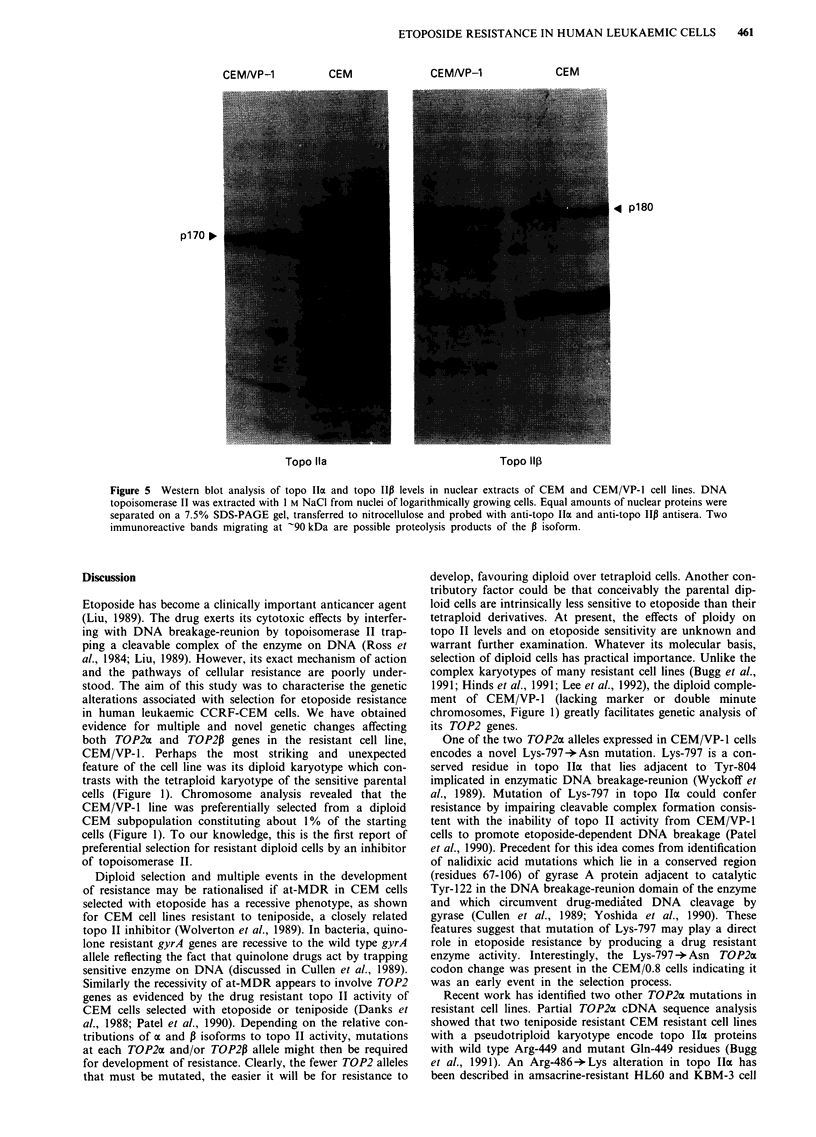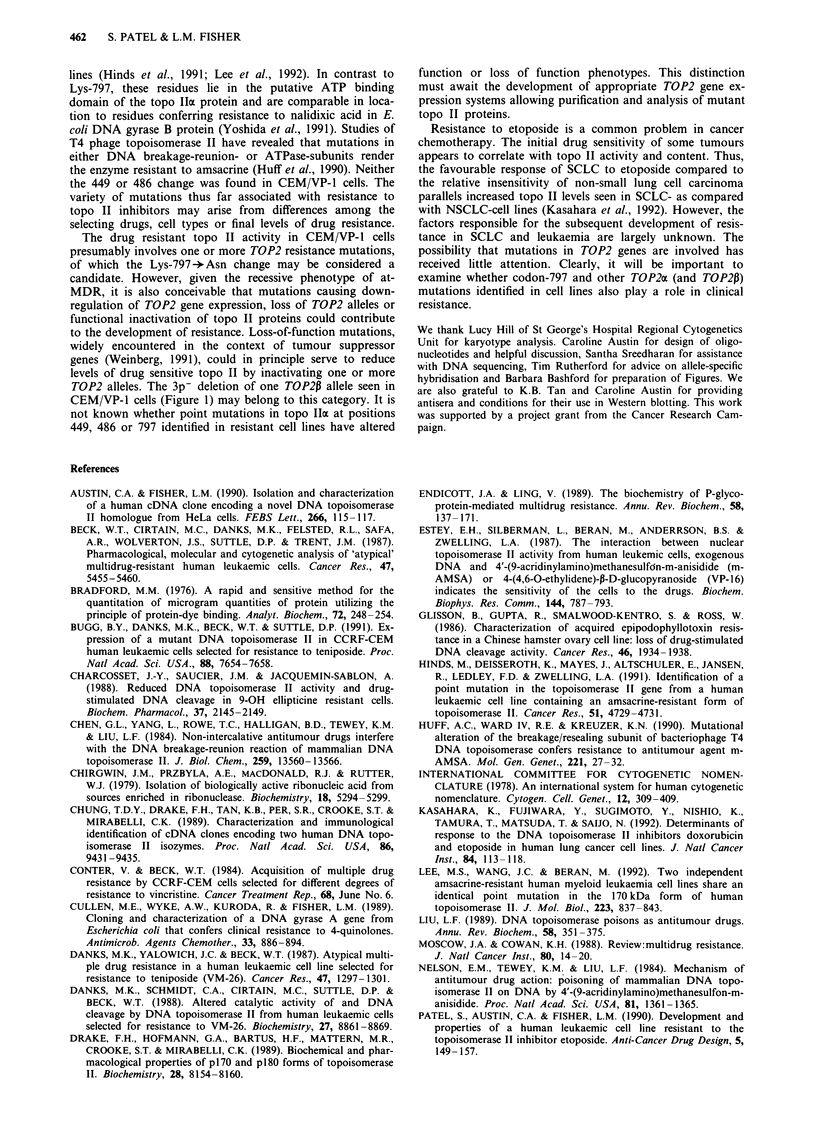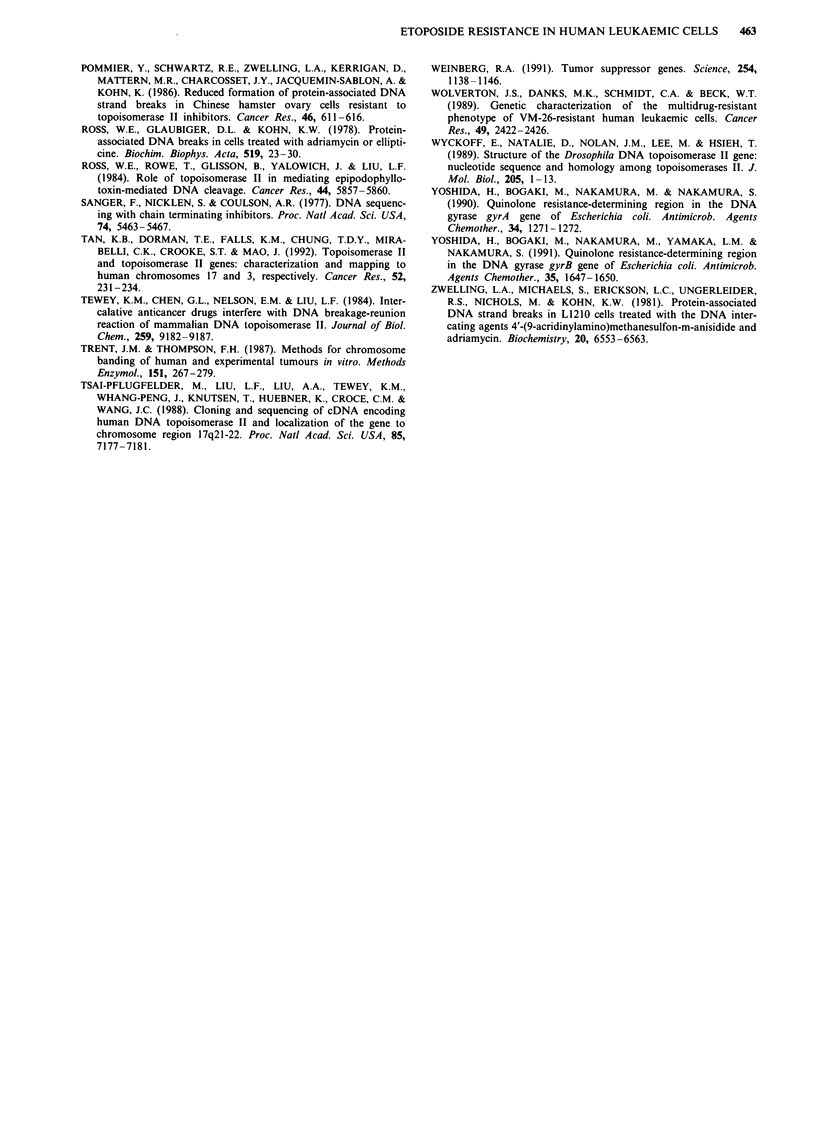# Novel selection and genetic characterisation of an etoposide-resistant human leukaemic CCRF-CEM cell line.

**DOI:** 10.1038/bjc.1993.87

**Published:** 1993-03

**Authors:** S. Patel, L. M. Fisher

**Affiliations:** Department of Cellular and Molecular Sciences, St George's Hospital Medical School, University of London, UK.

## Abstract

**Images:**


*Br. J. Cancer* (1993), **67**, 456–463 © Macmillan Press Ltd., 1993

### Novel selection and genetic characterisation of an etoposide-resistant human leukaemic CCRF-CEM cell line

S. Patel & L.M. Fisher

*Molecular Genetics Group, Department of Cellular and Molecular Sciences, St George's Hospital Medical School, University of London, Cranmer Terrace, London SW17 0RE, UK.*

**Summary** We have studied the genetic alterations acquired during selection of a cloned human leukaemic cell line (CEM/VP-1) that is 15-fold more resistant to the anticancer topoisomerase II-inhibitor etoposide than parental CCRF-CEM cells. CEM/VP-1 cells exhibit an 'atypical MDR' phenotype: cross resistance to other topo II inhibitors (but not *Vinca* alkaloids) and expression of a drug-resistant topo II activity. Cytogenetic and molecular studies revealed that the cell line carried multiple genetic changes affecting *TOP2* genes encoding both topo IIα and β isoforms.

CEM/VP-1 was diploid, 47,XX,+20, and appears to have been preferentially selected from a 1% diploid subpopulation present in the tetraploid parental cells. The same chromosomal abnormalities were present in resistant and sensitive cells except for an acquired $3p^-$ change most likely deleting one *TOP2β* allele. PCR/DNA sequence analysis and allele-specific hybridisation showed that one of two *TOP2α* alleles expressed in CEM/VP-1 cells had acquired a Lys-797→Asn codon change. This mutation lies close to the catalytic Tyr-804 residue of the protein and may interfere with drug-induced trapping of the cleavable complex. Alternatively, it could exert a loss of function phenotype. CEM/VP-1 cells did not exhibit codon 449 or 486 *TOP2α* mutations in the ATP binding domain reported in two other resistant cell lines. Diploid selection and multiple changes observed in CEM/VP-1 cells appear to be consequences of the recessive phenotype of at-MDR. These results may be useful in approaching the mechanisms of clinical resistance.

Resistance to multiple antitumour agents is a major problem in cancer chemotherapy. Some tumours are resistant to primary therapy, others become resistant during treatment. Progress in understanding the mechanisms of multidrug resistance (MDR) has come largely from studies of cultured cell lines and has identified at least two distinct phenotypes, so-called 'classical' and 'atypical' MDR (Moscow & Cowan, 1988).

Cells exhibiting 'classical' MDR are resistant to a range of structurally unrelated lipophilic drugs including the *Vinca* alkaloids, anthracyclines, actinomycin D and colchicine (Endicott & Ling, 1989). Resistance arises from reduced intracellular drug levels due to expression of the transmembrane P-glycoprotein efflux pump, the product of the *MDR1* gene. In contrast, 'atypical MDR' (at-MDR) involves cross resistance to drugs that inhibit the replicative enzyme, DNA topoisomerase II (Danks *et al.*, 1987, 1988). This ATP-requiring dimeric protein is a structural component of metaphase chromosomes and is responsible for chromosome segregation *via* transient double strand DNA breaks (Liu, 1989). Topo II inhibiting drugs are thought to exert their cytotoxicity by trapping a 'cleavable complex' of the enzyme on DNA and include the epipodophyllotoxins, etoposide (VP-16) and teniposide (VM-26), which have become prominent antitumour agents effective against small cell lung carcinoma (SCLC), leukaemia, lymphoma and other neoplasms (Chen *et al.*, 1984; Nelson *et al.*, 1984; Ross *et al.*, 1978; Ross *et al.*, 1984; Tewey *et al.*, 1984; Zwelling *et al.*, 1981). Cells exhibiting at-MDR do not have alterations in sensitivity to *Vinca* alkaloids, P-glycoprotein levels or drug accumulation (Beck *et al.*, 1987; Danks *et al.*, 1987). Instead, their salient feature is reduced cleavable complex formation arising from decreased topo II activity levels and/or structural changes in the enzyme. One or both of these changes have been described in Chinese hamster ovary cells made resistant to teniposide or 9-hydroxyellipticine, HL60 cells made resistant to mAMSA and CEM cells resistant to teniposide and etoposide (Charcosset *et al.*, 1988; Danks *et al.*, 1987, 1988; Estey *et al.*, 1987; Glisson *et al.*, 1986; Patel *et al.*, 1990; Pommier *et al.*, 1986). Unlike the genetic dominance seen for *MDR1*-mediated resistance (Endicott & Ling, 1989), cell fusion experiments have shown that at-MDR is phenotypically recessive to drug sensitivity (Wolverton *et al.*, 1989).

The genetic basis underlying at-MDR is poorly understood. The situation is complicated by the recent identification in mammalian cells of two genetically distinct isoforms of topo II, termed α (p170) and β (p180) encoded by the *TOP2α* and *TOP2β* genes recently mapped to human chromosomes 17 and 3, respectively (Austin & Fisher, 1990; Chung *et al.*, 1989; Drake *et al.*, 1989; Tan *et al.*, 1992 and refs therein). These proteins are differentially regulated in the cell cycle and exhibit different sensitivities to topo II inhibitors *in vitro*. Two recent studies have uncovered candidate resistance mutations in the *TOP2α* cDNA of cell lines made resistant to amsacrine and teniposide (Bugg *et al.*, 1991; Hinds *et al.*, 1991; Lee *et al.*, 1992). The role of topo IIβ in resistance has yet to be examined fully.

To investigate genetic changes involved in at-MDR, we chose to study the acquisition of resistance to etoposide, one of the most used topo II directed antitumour drugs. We have developed and cloned a human leukaemic CCRF-CEM cell line, CEM/VP-1, derived by incremental selection for resistance to etoposide (Patel *et al.*, 1990). CEM/VP-1 cells are 15-fold more resistant to etoposide than parental cells and exhibit the hallmarks of at-MDR: cross resistance to other topo II inhibitors including doxorubicin, teniposide and m-AMSA but not to *Vinca* alkaloids; similar levels of topo II activity compared to parental cells but much reduced etoposide-mediated DNA cleavage by nuclear extracts; and an etoposide-resistant topo II decatenation activity in nuclear extracts (Patel *et al.*, 1990). The results indicate that a structural alteration in topo II contributes to the etoposide resistance of CEM/VP-1 cells.

In this paper, we use a combination of cytogenetic analysis, PCR/DNA sequencing and allele-specific hybridisation to characterise the CEM/VP-1 cell line and its *TOP2* alleles. We show that the cell line derives from a rare diploid population in the tetraploid parental CEM cells and present evidence for multiple genetic changes involving *TOP2α* and *TOP2β* genes.

Correspondence: L.M. Fisher.

Abbreviations: MDR, multidrug resistance; topo II, DNA topoisomerase II; *m*-AMSA or amsacrine, 4′-(9-acridinylamino)methanesulphon-*m*-anisidide; cDNA, complementary DNA; PCR, polymermase chain reaction, dNTPs; deoxynucleoside triphosphates. SCLC; small cell lung carcinoma.

Received 22 July 1992; and in revised form 14 October 1992.

### Materials and methods

### *Cell lines and cell culture*

The CCRF-CEM human leukaemic cell line kindly provided by Dr A.P. Johnstone (Department of Cellular and Molecular Sciences, St George's Hospital Medical School) was grown as stationary suspension cultures in RPMI 1640 medium containing 2 mM glutamine (Gibco BRL, Paisley, UK) and supplemented with 10% heat-inactivated foetal bovine serum (Imperial Laboratories, Hampshire, UK), penicillin (100 IU $ml^{-1}$) and streptomycin (100 $\mu g\ ml^{-1}$). Cells were maintained at a density of $1.5 \times 10^5$ to $2.5 \times 10^5$ by subculturing every 3–4 days and incubating at 37°C in an atmosphere of 95% air and 5% $CO_2$.

Selection of the drug-resistant CEM/VP-1 subline has been described previously (Patel *et al.*, 1990). Briefly, CCRF-CEM cells were cultured over several weeks in the presence of sublethal amounts of etoposide (80–160 nM). Resistant cells were then challenged intermittently and repeatedly (3–4 times) with each incremental concentration (0.32, 0.8, 1.0 and 1.6 $\mu$M) of etoposide (Bristol-Myers Co, Syracuse, NY), allowing for recovery of cultured cells during the procedure. Cell counts and cell viability were assessed using a haemocytometer and Trypan Blue dye exclusion, respectively. Cells resistant to the different levels of etoposide were subcultured and stocks were frozen in liquid nitrogen. Subsequently, cells selected at 0.8 $\mu$M (but not 0.32 $\mu$M) drug could be grown up from frozen stocks and were designated CEM/0.8. The CEM/VP-1 subline was derived from cells able to grow in 1.6 $\mu$M etoposide and was obtained by cloning in soft agarose in the absence of drug (Patel *et al.*, 1990). Drug cytotoxicities were determined by the method of Conter and Beck, 1984 and were expressed as $IC_{50}$ values, the drug concentration that inhibits cell growth by 50% compared to control cells. The resistant phenotype of CEM/VP-1 cells appeared to be stable: cells could be grown over long periods in the absence of drug without loss of resistance.

### *Karyotype analysis*

Cytogenetic analysis of parental CEM and drug-resistant sublines was performed at the same time using the standard G-banding technique (Trent & Thompson, 1987). Logarithmically growing cultures, were prepared for chromosome analysis by pretreatment for 4 h with colchicine to arrest cells in mitosis. The cells were collected by gentle centrifugation, resuspended in a hypotonic 0.075 M potassium chloride solution, recovered by centrifugation and fixed in methanol:acetic acid (3:1). Slides were prepared by air drying. The spreads were trypsinised in 0.1% trypsin for 2 min after which they were stained with Giemsa. Karyotypes are described according to the International System for Cytogenetic Nomenclature, 1978.

### *PCR/DNA sequence analysis of* TOP2α *cDNA from cell lines*

Total cellular RNA was isolated from CEM, CEM/0.8 and CEM/VP-1 cells ($1.5 \times 10^7$ cells) by the guanidinium thiocyanate method (Chirgwin *et al.*, 1979) and used for first strand *TOP2α* cDNA synthesis. RNA (2 $\mu$g) was primed for cDNA synthesis with one of three 18-mer *TOP2α* antisense oligonucleotides: oligo 2, 5′ TATGAGAAGCTTCTCGAA (nucleotide positions 1492–1475); oligo 4, 5′ TAGCCTG-GTACCAAACTG (2379–2362); or oligo 6, 5′ CTTAGC-CTGCAGAGTTCA (3440–3423) (nucleotide numbering system based on the *TOP2α* sequence of Tsai-Pflugfelder *et al.*, 1988, which labels base 1 as the A of the ATG initiation codon). First strand cDNA synthesis was carried out using moloney murine leukaemia virus reverse transcriptase (Gibco-BRL, Paisley, Scotland) according to the manufacturer's instructions (20 $\mu$l total volume). Polymerase chain reactions were carried out using these cDNA products primed with oligos 2, 4 or 6 and the sense oligonucleotides: 1, 5′ ATGGAAGTGTCACCATTG (1–18); 3, 5′ TTCGAG-AAGCTTCTCATA (1475–1492); or 5, 5′ CAGTTTG-GTACCAGGCTA (2362–2379) (Figure 2). Each first strand cDNA product (4 $\mu$l) was added to PCR reaction mixtures containing 50 pmol of each oligonucleotide primer, 200 $\mu$M (each) dNTP and 2.5 U *Taq* polymerase (Cambio, Cambridge, UK) in 100 $\mu$l reaction buffer (50 mM KCl/10 mM Tris-HCl/1.5 mM $MgCl_2$/0.1 mg $ml^{-1}$ gelatin, pH 8.0). PCR conditions were: 95°C, 1 min; 50°C, 1 min; 74°C, 3 min; 30 cycles. PCR products were precipitated with ethanol, digested with restriction enzymes (Northumbria Biologicals Ltd., Northumberland, UK) and the resulting fragments ligated into appropriately cut M13mp18 or pBluescript II $S/K^+$ vectors (Stratagene Ltd., Cambridge, UK) prior to transformation of *E. coli* XL-1 Blue, as recommended by Amersham International (Little Chalfont, Bucks, UK). DNA was sequenced by the dideoxy chain termination method (Sanger *et al.*, 1977) using Sequenase version 2.0 (US Biochemicals) and [α-$^{35}$S]dATP (Amersham). DNA sequencing reactions were run on 6% denaturing polyacrylamide gels which were fixed in 10% acetic acid-10% methanol for 30 min, dried and autoradiographed on Fuji X-ray film. DNA sequences were analysed using the PC/Gene software package (IntelliGenetics, Mountain View, California).

### *Allele-specific oligonucleotide hybridisation to* TOP2α *cDNA*

PCR products derived from CEM, CEM/0.8 and CEM/VP-1 cDNA using oligo pair 5/6 (Figure 2) were electrophoresed on a 0.8% agarose gel, blotted on to nylon membranes and prehybridised for 4 h at 45°C in 5 × SSPE (1 × SSPE is 10 mM sodium phosphate, pH 7.2/0.18 M NaCl/1 mM $Na_3$ EDTA, pH 7.4) containing 7% SDS, 5 × Denhardt's reagent and 100 $\mu g\ ml^{-1}$ salmon sperm DNA. Hybridisation was overnight at 45°C in the same buffer containing either 5′$^{32}$P-labelled oligo S (5′ AGCAGAATCCTTGCCACCAT) or R (5′ AGCAGAATCATTGCCACCAT). Filters were washed at room temperature for 20 min in 2 × SSPE/0.1% SDS and then for 1 h at 55°C in 5 × SSPE/0.1% SDS. Filters were subjected to a final wash in 5 × SSPE/0.1% SDS for 10 min at either 60°C (oligo S probe) or 58°C (oligo R probe), respectively. (These temperatures were the calculated $T_m$ s for each oligonucleotide). Autoradiography was at room temperature for 1–2 h using Amersham Hyperfilm.

### *Immunological detection of topo IIα and IIβ*

Nuclear proteins (100 $\mu$g), prepared from logarithmically-growing CEM cell lines (Danks *et al.*, 1988), were separated by electrophoresis on 7.5% sodium dodecyl sulphate-polyacrylamide gels (samples to be analysed for topo IIα and β were boiled for 2 min or heated at 68°C for 5 min respectively, prior to loading). Proteins were transferred to nitrocellulose membranes which were blocked overnight in TBST buffer (10 mM Tris-HCl, pH 7.5, 0.15 M NaCl, 0.05% Tween) containing 1% BSA (the latter was omitted for filters to be probed for topo IIβ). Filters were probed by incubation for 2 h at room temperature with an affinity-purified rabbit anti-topo IIα antiserum (kindly provided by Dr F. Drake, SmithKline Beecham, King of Prussia, USA) diluted 1:1000 in TBST, Topo IIβ was detected by incubating membranes for 2 h at 37°C with an affinity purified anti-topo IIβ antiserum (raised against a synthetic topo IIβ peptide sequence by Dr Caroline Austin of this laboratory) diluted 1:200 in TBST containing 0.1% SDS. Blots were washed three times in TBST for 3–5 min each and then incubated for 2 h with goat anti-rabbit IgG alkaline phosphatase conjugate (Sigma Co, Poole, UK)) diluted 1:1000. Filters were washed three times with TBST for 5 min prior to development of colour reactions using nitroblue tetrazolium and BCIP (5-bromo-4-chloro-3-indoyl phosphate) (Promega) according to the manufacturer's instructions. Sizes of immunoreactive bands were determined by running protein markers alongside and whose position could be located on the nitrocellulose filter by staining with Amido black.

**Figure 1** Cytogenetic analysis of CEM **a**, and CEM/VP-1 **b**, cells by the G banding method. **a**, Karyotype of the drug-sensitive CEM cell line: arrows denote structural alterations del(8) (p11-pter), del(9) (p13-pter), t(9,?) (p22,?) and + 20. **b**, CEM/VP-1 cells were diploid and exhibited the same chromosome abnormalities with an additional del(3) (p13-pter) alteration.

*Other methods*

Oligonucleotides were made on a Cyclone Plus DNA synthesiser (Milligen/Biosearch, Ltd) using phosphoramidite chemistry. Protein concentrations were determined by the method of Bradford, 1976 using bovine plasma albumin as a standard.

### Results

*Preferential selection of CEM/VP-1 from a diploid subpopulation: an acquired chromosome 3p deletion affects one* TOP2β *allele*

Chromosome banding analysis was carried out on 20 metaphase spreads from both CEM and CEM/VP-1 cell lines (Figure 1). CEM cells were largely tetraploid ($n = 94$) and carried extra copies of chromosome 20 (Figure 1a). Survey of a large number of spreads revealed that approximately 1% of CEM cells were diploid ($n = 47$) with chromosome 20 in extra copy. Three structural abnormalities were identified for the diploid and tetraploid CEM cells: del(8) (p11-pter), del(9) (p13-pter) and t(9;?) (p22;?). Surprisingly, the CEM/VP-1 cell line was found to be diploid ($n = 47$, +20), exhibiting the same numerical and structural abnormalities as CEM cells (Figure 1b). However, one homologue of chromosome 3 in the CEM/VP-1 cell line had acquired the additional abnormality, del(3) (p13-pter). This 3p region was not visible elsewhere in the chromosome spread consistent with its deletion or a complex rearrangement. No marker chromosomes, double minutes or homogenously staining regions were observed in any of the metaphase spreads examined for either parental or resistant cells. The results establish that the etoposide-resistant cell line has two copies of chromosome 17 on which maps the *TOP2* allele (17p21-22) coding for p170 topoisomerase II (Tsai-Pflugfelder *et al.*, 1988), and most likely only one allele for the p180 isoform, provisionally mapped at 3p24-25 (Tan *et al.*, 1992).

The resistant cell line appeared to have been selected from the 1% diploid subgroup of CEM cells (from which the tetraploid population presumably also originated during extended culture of the CEM line). This view is supported by karyotype analysis of CEM/0.8 cells which were intermediates in the stepwise selection of CEM/VP-1 and were ~10-fold more resistant to etoposide than parental cells (data not shown). Diploid cells ($n = 47$) accounted for 87 of the 100 CEM/0.8 metaphases examined, with the remainder being tetraploid (data not shown). Each type of spread showed the same structural abnormalities as observed in the respective diploid and tetraploid CEM cells. However, both number 3 chromosomes appeared normal in the diploid CEM/0.8 cells suggesting that the del(3) (p13-pter) change observed in CEM/VP-1 cells occurred later in the selection process.

*A novel* TOP2α *mutation is acquired during etoposide selection*

Previously, we showed that topo II activity in CEM/VP-1 nuclear extracts was not inhibited by etoposide and did not mediate drug-induced DNA cleavage *in vitro* (Patel *et al.*, 1990). These properties are reminiscent of 4-quinolone resistance in *Escherichia coli* strains arising from mutation of DNA gyrase, a bacterial DNA topoisomerase II. Mutations in gyrase A protein adjacent to the catalytic Tyr-122 residue engaged in DNA breakage-reunion generate a drug resistant supercoiling activity and abolish quinolone induced DNA breakage by the complex (Cullen *et al.*, 1989; Yoshida *et al.*, 1990). The analogy with the gyrase system led us to search for potential etoposide resistance mutations in the p170 *TOP2* cDNA of the CEM/VP-1 cell line, focusing on the region surrounding codon-804 which specifies the tyrosine residue implicated in transient DNA breakage-reunion (Tsai-Pflugfelder *et al.*, 1988; Wyckoff *et al.*, 1989).

PCR products A-C, spanning the p170 *TOP2* cDNA sequence from CEM and CEM/VP-1 cells, were obtained by first strand cDNA/PCR amplification from total cellular RNA using three sets of oligonucleotide primers, 1/2, 3/4 and 5/6 (Figure 2). These 1.5, 0.88 and 1.0 kb products were digested with *Bam*HI/*Hind*III, *Hind*III/*Kpn*I and *Kpn*I/*Bam*HI, respectively and cloned into M13mp18 or Bluescript allowing DNA sequence analysis. DNA sequence obtained from the *Kpn*I ends of fragments B and C covered codons 740-840 including the catalytic Tyr-804 codon. Four independent clones of fragment C derived from the CEM/VP-1 cell line and nine clones from sensitive CEM cells were analysed and compared to the HeLa p170 cDNA sequence (Tsai-Pflugfelder *et al.*, 1988). Three of the four CEM/VP-1 clones carried a single base change, a G→T transversion at nucleotide position 2391 (Figure 3a), corresponding to a Lys-797→Asn substitution at the protein level. The fourth clone was identical in sequence to HeLa p170 cDNA in this region (data not shown). None of the nine CEM-drived clones differed in sequence from that of the HeLa p170 cDNA (Figure 3a). Thus, the diploid CEM/VP-1 cells appear to express two *TOP2α* alleles encoding wild-type and mutant 797 protein residues.

*Allele-specific hybridisation*

Expression of *TOP2α* alleles in the resistant CEM/VP-1 cells was further analysed by oligonucleotide hybridisation (Figures 3b and 4). Allele-specific antisense oligonucleotides

**Figure 2** Restriction map of the full length human p170 *TOP2* cDNA (heavy line) showing regions **a–c**, amplified by polymerase chain reaction from a first strand cDNA derived from CEM and CEM/VP-1 cell RNA. Restriction sites for *Bam*HI, *Hind*III and *Kpn*I are indicated. The A of the ATG initiation codon and the T of the TAA stop codon are labelled as nucleotide positions 1 and 4591, respectively, as in Tsai-Pflugfelder *et al.*, 1988. Antisense oligos 2, 4 and 6 were used for first strand cDNA synthesis; oligos 1/2, 3/4 and 5/6 were used in PCR reactions to obtain products A-C (see text for details). Open triangle shows the position of codon 804 which specifies the catalytic Tyr residue involved in transient DNA breakage-reunion. Filled circles locate codons 449, 486; asterisk, codon 797. Lines below cDNA indicate DNA sequence determined for PCR products. Bar denotes 1 kilobase pairs.

b

```

                   T

                   ¦

CAT GGT GGC AAG GAT TCT GCT AGT CCA CGA TAC ATC TTT

His Gly Gly Lys Asp Ser Ala Ser Pro Arg TYR Ile Phe

                   ¦

            Asn-797   (CEM/VP-1)       804

```

```

TA CCA CCG TTC CTA AGA CGA 5'       Oligo S

TA CCA CCG TTA CTA AGA CGA 5'       Oligo R

2381                     2400

```

(In Oligo R, the A of TTA is underlined.)

**Figure 3** Identification of a Lys-797→Asn codon change in the p170 *TOP2* cDNA from etoposide-resistant CEM/VP-1 cells. PCR product C was digested with *Kpn*I and *Bam*HI (Figure 2), subcloned into pBluescript II S/K$^+$ and sequenced directly by the dideoxy chain termination method (Sanger *et al.*, 1977). **a**, Sequencing gel showing a region of coding strand DNA encompassing codon 797. Asterisk indicates a G→T change at nucleotide position 2391 in the resistant CEM/VP-1 cDNA that is absent in the *TOP2α* coding sequence of parental CEM cells. **b**, CEM/VP-1 mutation in p170 topo II lies close to the catalytic tyrosine residue 804. Diagram also shows the sequence of two antisense oligonucleotides S and R designed to detect normal and mutant alleles, respectively. The base change in the mutant oligo R is underlined.

were synthesised corresponding to normal (oligo S) or mutant CEM/VP-1 (oligo R) sequences encompassing codon 797 (Figure 3b). Equal amounts (approximately 0.4 μg) of PCR product C made from CEM and CEM/VP-1 cDNA were electrophoresed in a 0.8% agarose gel, blotted on to nylon filters and probed with 5′ end-labelled oligo S or oligo R. PCR product from CEM/VP-1 cells hybridised to both mutant and normal oligonucleotide probes whereas that from

**Figure 4** Mutant (Lys-797→Asn) p170 *TOP2* allele is expressed in the etoposide-resistant CEM/VP-1 cell line, but not in sensitive CEM cells. Allele specific oligonucleotide hybridisation to PCR product C derived from cDNA of drug sensitive and etoposide-resistant CEM/VP-1 cells. PCR products were run on a 0.8% agarose gel, transferred to nylon filters and hybridised to 5′ $^{32}$P-labelled oligo S (**a**) or oligo R (**b**) (see Figure 3 legend).

CEM cells hybridised only with oligo S (Figure 4). These results confirm the expression of one mutant *TOP2α* allele in etoposide-resistant cells and its absence in drug sensitive parental CEM cells.

By using the same approach, it was found that the codon 797 *TOPα* mutation was present in CEM/0.8 cells, intermediates in the isolation of CEM/VP-1 (data not shown). Thus, mutation of Lys-797 in topo IIα appears to be an early event in selection for etoposide resistance.

### *Absence of codon 449 and 486* TOP2α *mutations*

DNA sequence was determined for four CEM/VP-1 clones of PCR product A corresponding to *TOP2α* codons 381–495. This region contains codons 449 and 486 which are known to be altered in m-AMSA resistant HL60 and teniposide resistant CEM cell lines (Bugg *et al.*, 1991; Hinds *et al.*, 1991; Lee *et al.*, 1992). However, each CEM/VP-1 cDNA sequence was identical to that reported for HeLa *TOP2α* cDNA (Tsai-Pflugfelder *et al.*, 1988): there was no evidence of coding changes in this region (data not shown).

### *Topo IIα and IIβ protein levels*

Western blot analysis revealed that CEM cells express both α and β isoforms of topo II and that roughly similar levels (to within 2-fold) were present in CEM/VP-1 cells (Figure 5). These results parallel our previous work showing that resistant cells had no more than a 2-fold reduction in topoisomerase II activity (Patel *et al.*, 1990). Thus, although the effects of ploidy and of the relative contributions of α and β isoforms to topo II activity are not known, etoposide resistance in the CEM/VP-1 cell line did not appear to involve the elimination or marked down-regulation of either topo II isoform.

**Figure 5** Western blot analysis of topo IIα and topo IIβ levels in nuclear extracts of CEM and CEM/VP-1 cell lines. DNA topoisomerase II was extracted with 1 M NaCl from nuclei of logarithmically growing cells. Equal amounts of nuclear proteins were separated on a 7.5% SDS-PAGE gel, transferred to nitrocellulose and probed with anti-topo IIα and anti-topo IIβ antisera. Two immunoreactive bands migrating at ~90 kDa are possible proteolysis products of the β isoform.

### Discussion

Etoposide has become a clinically important anticancer agent (Liu, 1989). The drug exerts its cytotoxic effects by interfering with DNA breakage-reunion by topoisomerase II trapping a cleavable complex of the enzyme on DNA (Ross *et al.*, 1984; Liu, 1989). However, its exact mechanism of action and the pathways of cellular resistance are poorly understood. The aim of this study was to characterise the genetic alterations associated with selection for etoposide resistance in human leukaemic CCRF-CEM cells. We have obtained evidence for multiple and novel genetic changes affecting both *TOP2α* and *TOP2β* genes in the resistant cell line, CEM/VP-1. Perhaps the most striking and unexpected feature of the cell line was its diploid karyotype which contrasts with the tetraploid karyotype of the sensitive parental cells (Figure 1). Chromosome analysis revealed that the CEM/VP-1 line was preferentially selected from a diploid CEM subpopulation constituting about 1% of the starting cells (Figure 1). To our knowledge, this is the first report of preferential selection for resistant diploid cells by an inhibitor of topoisomerase II.

Diploid selection and multiple events in the development of resistance may be rationalised if at-MDR in CEM cells selected with etoposide has a recessive phenotype, as shown for CEM cell lines resistant to teniposide, a closely related topo II inhibitor (Wolverton *et al.*, 1989). In bacteria, quinolone resistant *gyrA* genes are recessive to the wild type *gyrA* allele reflecting the fact that quinolone drugs act by trapping sensitive enzyme on DNA (discussed in Cullen *et al.*, 1989). Similarly the recessivity of at-MDR appears to involve *TOP2* genes as evidenced by the drug resistant topo II activity of CEM cells selected with etoposide or teniposide (Danks *et al.*, 1988; Patel *et al.*, 1990). Depending on the relative contributions of α and β isoforms to topo II activity, mutations at each *TOP2α* and/or *TOP2β* allele might then be required for development of resistance. Clearly, the fewer *TOP2* alleles that must be mutated, the easier it will be for resistance to develop, favouring diploid over tetraploid cells. Another contributory factor could be that conceivably the parental diploid cells are intrinsically less sensitive to etoposide than their tetraploid derivatives. At present, the effects of ploidy on topo II levels and on etoposide sensitivity are unknown and warrant further examination. Whatever its molecular basis, selection of diploid cells has practical importance. Unlike the complex karyotypes of many resistant cell lines (Bugg *et al.*, 1991; Hinds *et al.*, 1991; Lee *et al.*, 1992), the diploid complement of CEM/VP-1 (lacking marker or double minute chromosomes, Figure 1) greatly facilitates genetic analysis of its *TOP2* genes.

One of the two *TOP2α* alleles expressed in CEM/VP-1 cells encodes a novel Lys-797→Asn mutation. Lys-797 is a conserved residue in topo IIα that lies adjacent to Tyr-804 implicated in enzymatic DNA breakage-reunion (Wyckoff *et al.*, 1989). Mutation of Lys-797 in topo IIα could confer resistance by impairing cleavable complex formation consistent with the inability of topo II activity from CEM/VP-1 cells to promote etoposide-dependent DNA breakage (Patel *et al.*, 1990). Precedent for this idea comes from identification of nalidixic acid mutations which lie in a conserved region (residues 67-106) of gyrase A protein adjacent to catalytic Tyr-122 in the DNA breakage-reunion domain of the enzyme and which circumvent drug-mediated DNA cleavage by gyrase (Cullen *et al.*, 1989; Yoshida *et al.*, 1990). These features suggest that mutation of Lys-797 may play a direct role in etoposide resistance by producing a drug resistant enzyme activity. Interestingly, the Lys-797→Asn *TOP2α* codon change was present in the CEM/0.8 cells indicating it was an early event in the selection process.

Recent work has identified two other *TOP2α* mutations in resistant cell lines. Partial *TOP2α* cDNA sequence analysis showed that two teniposide resistant CEM resistant cell lines with a pseudotriploid karyotype encode topo IIα proteins with wild type Arg-449 and mutant Gln-449 residues (Bugg *et al.*, 1991). An Arg-486→Lys alteration in topo IIα has been described in amsacrine-resistant HL60 and KBM-3 cell lines (Hinds *et al.*, 1991; Lee *et al.*, 1992). In contrast to Lys-797, these residues lie in the putative ATP binding domain of the topo IIα protein and are comparable in location to residues conferring resistance to nalidixic acid in *E. coli* DNA gyrase B protein (Yoshida *et al.*, 1991). Studies of T4 phage topoisomerase II have revealed that mutations in either DNA breakage-reunion- or ATPase-subunits render the enzyme resistant to amsacrine (Huff *et al.*, 1990). Neither the 449 or 486 change was found in CEM/VP-1 cells. The variety of mutations thus far associated with resistance to topo II inhibitors may arise from differences among the selecting drugs, cell types or final levels of drug resistance.

The drug resistant topo II activity in CEM/VP-1 cells presumably involves one or more *TOP2* resistance mutations, of which the Lys-797→Asn change may be considered a candidate. However, given the recessive phenotype of at-MDR, it is also conceivable that mutations causing down-regulation of *TOP2* gene expression, loss of *TOP2* alleles or functional inactivation of topo II proteins could contribute to the development of resistance. Loss-of-function mutations, widely encountered in the context of tumour suppressor genes (Weinberg, 1991), could in principle serve to reduce levels of drug sensitive topo II by inactivating one or more *TOP2* alleles. The $3p^-$ deletion of one *TOP2β* allele seen in CEM/VP-1 cells (Figure 1) may belong to this category. It is not known whether point mutations in topo IIα at positions 449, 486 or 797 identified in resistant cell lines have altered function or loss of function phenotypes. This distinction must await the development of appropriate *TOP2* gene expression systems allowing purification and analysis of mutant topo II proteins.

Resistance to etoposide is a common problem in cancer chemotherapy. The initial drug sensitivity of some tumours appears to correlate with topo II activity and content. Thus, the favourable response of SCLC to etoposide compared to the relative insensitivity of non-small lung cell carcinoma parallels increased topo II levels seen in SCLC- as compared with NSCLC-cell lines (Kasahara *et al.*, 1992). However, the factors responsible for the subsequent development of resistance in SCLC and leukaemia are largely unknown. The possibility that mutations in *TOP2* genes are involved has received little attention. Clearly, it will be important to examine whether codon-797 and other *TOP2α* (and *TOP2β*) mutations identified in cell lines also play a role in clinical resistance.

We thank Lucy Hill of St George's Hospital Regional Cytogenetics Unit for karyotype analysis. Caroline Austin for design of oligonucleotides and helpful discussion, Santha Sreedharan for assistance with DNA sequencing, Tim Rutherford for advice on allele-specific hybridisation and Barbara Bashford for preparation of Figures. We are also grateful to K.B. Tan and Caroline Austin for providing antisera and conditions for their use in Western blotting. This work was supported by a project grant from the Cancer Research Campaign.

### References

AUSTIN, C.A. & FISHER, L.M. (1990). Isolation and characterization of a human cDNA clone encoding a novel DNA topoisomerase II homologue from HeLa cells. *FEBS Lett.*, **266**, 115-117.

BECK, W.T., CIRTAIN, M.C., DANKS, M.K., FELSTED, R.L., SAFA, A.R., WOLVERTON, J.S., SUTTLE, D.P. & TRENT, J.M. (1987). Pharmacological, molecular and cytogenetic analysis of 'atypical' multidrug-resistant human leukaemic cells. *Cancer Res.*, **47**, 5455-5460.

BRADFORD, M.M. (1976). A rapid and sensitive method for the quantitation of microgram quantities of protein utilizing the principle of protein-dye binding. *Analyt. Biochem.*, **72**, 248-254.

BUGG, B.Y., DANKS, M.K., BECK, W.T. & SUTTLE, D.P. (1991). Expression of a mutant DNA topoisomerase II in CCRF-CEM human leukaemic cells selected for resistance to teniposide. *Proc. Natl Acad. Sci. USA*., **88**, 7654-7658.

CHARCOSSET, J.-Y., SAUCIER, J.M. & JACQUEMIN-SABLON, A. (1988). Reduced DNA topoisomerase II activity and drug-stimulated DNA cleavage in 9-OH ellipticine resistant cells. *Biochem. Pharmacol.*, **37**, 2145-2149.

CHEN, G.L., YANG, L., ROWE, T.C., HALLIGAN, B.D., TEWEY, K.M. & LIU, L.F. (1984). Non-intercalative antitumour drugs interfere with the DNA breakage-reunion reaction of mammalian DNA topoisomerase II. *J. Biol. Chem.*, **259**, 13560-13566.

CHIRGWIN, J.M., PRZBYLA, A.E., MACDONALD, R.J. & RUTTER, W.J. (1979). Isolation of biologically active ribonucleic acid from sources enriched in ribonuclease. *Biochemistry*, **18**, 5294-5299.

CHUNG, T.D.Y., DRAKE, F.H., TAN, K.B., PER, S.R., CROOKE, S.T. & MIRABELLI, C.K. (1989). Characterization and immunological identification of cDNA clones encoding two human DNA topoisomerase II isozymes. *Proc. Natl Acad. Sci. USA*, **86**, 9431-9435.

CONTER, V. & BECK, W.T. (1984). Acquisition of multiple drug resistance by CCRF-CEM cells selected for different degrees of resistance to vincristine. *Cancer Treatment Rep.*, **68**, June No. 6.

CULLEN, M.E., WYKE, A.W., KURODA, R. & FISHER, L.M. (1989). Cloning and characterization of a DNA gyrase A gene from *Escherichia coli* that confers clinical resistance to 4-quinolones. *Antimicrob. Agents Chemother.*, **33**, 886-894.

DANKS, M.K., YALOWICH, J.C. & BECK, W.T. (1987). Atypical multiple drug resistance in a human leukaemic cell line selected for resistance to teniposide (VM-26). *Cancer Res.*, **47**, 1297-1301.

DANKS, M.K., SCHMIDT, C.A., CIRTAIN, M.C., SUTTLE, D.P. & BECK, W.T. (1988). Altered catalytic activity of and DNA cleavage by DNA topoisomerase II from human leukaemic cells selected for resistance to VM-26. *Biochemistry*, **27**, 8861-8869.

DRAKE, F.H., HOFMANN, G.A., BARTUS, H.F., MATTERN, M.R., CROOKE, S.T. & MIRABELLI, C.K. (1989). Biochemical and pharmacological properties of p170 and p180 forms of topoisomerase II. *Biochemistry*, **28**, 8154-8160.

ENDICOTT, J.A. & LING, V. (1989). The biochemistry of P-glycoprotein-mediated multidrug resistance. *Annu. Rev. Biochem.*, **58**, 137-171.

ESTEY, E.H., SILBERMAN, L., BERAN, M., ANDERRSON, B.S. & ZWELLING, L.A. (1987). The interaction between nuclear topoisomerase II activity from human leukemic cells, exogenous DNA and 4'-(9-acridinylamino)methanesulfon-m-anisidide (m-AMSA) or 4-(4,6-O-ethylidene)-β-D-glucopyranoside (VP-16) indicates the sensitivity of the cells to the drugs. *Biochem. Biophys. Res. Comm.*, **144**, 787-793.

GLISSON, B., GUPTA, R., SMALWOOD-KENTRO, S. & ROSS, W. (1986). Characterization of acquired epipodophyllotoxin resistance in a Chinese hamster ovary cell line: loss of drug-stimulated DNA cleavage activity. *Cancer Res.*, **46**, 1934-1938.

HINDS, M., DEISSEROTH, K., MAYES, J., ALTSCHULER, E., JANSEN, R., LEDLEY, F.D. & ZWELLING, L.A. (1991). Identification of a point mutation in the topoisomerase II gene from a human leukaemic cell line containing an amsacrine-resistant form of topoisomerase II. *Cancer Res.*, **51**, 4729-4731.

HUFF, A.C., WARD IV, R.E. & KREUZER, K.N. (1990). Mutational alteration of the breakage/resealing subunit of bacteriophage T4 DNA topoisomerase confers resistance to antitumour agent m-AMSA. *Mol. Gen. Genet.*, **221**, 27-32.

INTERNATIONAL COMMITTEE FOR CYTOGENETIC NOMENCLATURE (1978). An international system for human cytogenetic nomenclature. *Cytogen. Cell. Genet.*, **12**, 309-409.

KASAHARA, K., FUJIWARA, Y., SUGIMOTO, Y., NISHIO, K., TAMURA, T., MATSUDA, T. & SAIJO, N. (1992). Determinants of response to the DNA topoisomerase II inhibitors doxorubicin and etoposide in human lung cancer cell lines. *J. Natl Cancer Inst.*, **84**, 113-118.

LEE, M.S., WANG, J.C. & BERAN, M. (1992). Two independent amsacrine-resistant human myeloid leukaemia cell lines share an identical point mutation in the 170 kDa form of human topoisomerase II. *J. Mol. Biol.*, **223**, 837-843.

LIU, L.F. (1989). DNA topoisomerase poisons as antitumour drugs. *Annu. Rev. Biochem.*, **58**, 351-375.

MOSCOW, J.A. & COWAN, K.H. (1988). Review:multidrug resistance. *J. Natl Cancer Inst.*, **80**, 14-20.

NELSON, E.M., TEWEY, K.M. & LIU, L.F. (1984). Mechanism of antitumour drug action: poisoning of mammalian DNA topoisomerase II on DNA by 4'-(9-acridinylamino)methanesulfon-m-anisidide. *Proc. Natl Acad. Sci. USA*, **81**, 1361-1365.

PATEL, S., AUSTIN, C.A. & FISHER, L.M. (1990). Development and properties of a human leukaemic cell line resistant to the topoisomerase II inhibitor etoposide. *Anti-Cancer Drug Design*, **5**, 149-157.

POMMIER, Y., SCHWARTZ, R.E., ZWELLING, L.A., KERRIGAN, D., MATTERN, M.R., CHARCOSSET, J.Y., JACQUEMIN-SABLON, A. & KOHN, K. (1986). Reduced formation of protein-associated DNA strand breaks in Chinese hamster ovary cells resistant to topoisomerase II inhibitors. *Cancer Res.*, **46**, 611–616.

ROSS, W.E., GLAUBIGER, D.L. & KOHN, K.W. (1978). Protein-associated DNA breaks in cells treated with adriamycin or ellipticine. *Biochim. Biophys. Acta*, **519**, 23–30.

ROSS, W.E., ROWE, T., GLISSON, B., YALOWICH, J. & LIU, L.F. (1984). Role of topoisomerase II in mediating epipodophyllotoxin-mediated DNA cleavage. *Cancer Res.*, **44**, 5857–5860.

SANGER, F., NICKLEN, S. & COULSON, A.R. (1977). DNA sequencing with chain terminating inhibitors. *Proc. Natl Acad. Sci. USA*, **74**, 5463–5467.

TAN, K.B., DORMAN, T.E., FALLS, K.M., CHUNG, T.D.Y., MIRABELLI, C.K., CROOKE, S.T. & MAO, J. (1992). Topoisomerase II and topoisomerase II genes: characterization and mapping to human chromosomes 17 and 3, respectively. *Cancer Res.*, **52**, 231–234.

TEWEY, K.M., CHEN, G.L., NELSON, E.M. & LIU, L.F. (1984). Intercalative anticancer drugs interfere with DNA breakage-reunion reaction of mammalian DNA topoisomerase II. *Journal of Biol. Chem.*, **259**, 9182–9187.

TRENT, J.M. & THOMPSON, F.H. (1987). Methods for chromosome banding of human and experimental tumours *in vitro*. *Methods Enzymol.*, **151**, 267–279.

TSAI-PFLUGFELDER, M., LIU, L.F., LIU, A.A., TEWEY, K.M., WHANG-PENG, J., KNUTSEN, T., HUEBNER, K., CROCE, C.M. & WANG, J.C. (1988). Cloning and sequencing of cDNA encoding human DNA topoisomerase II and localization of the gene to chromosome region 17q21-22. *Proc. Natl Acad. Sci. USA*, **85**, 7177–7181.

WEINBERG, R.A. (1991). Tumor suppressor genes. *Science*, **254**, 1138–1146.

WOLVERTON, J.S., DANKS, M.K., SCHMIDT, C.A. & BECK, W.T. (1989). Genetic characterization of the multidrug-resistant phenotype of VM-26-resistant human leukaemic cells. *Cancer Res.*, **49**, 2422–2426.

WYCKOFF, E., NATALIE, D., NOLAN, J.M., LEE, M. & HSIEH, T. (1989). Structure of the *Drosophila* DNA topoisomerase II gene: nucleotide sequence and homology among topoisomerases II. *J. Mol. Biol.*, **205**, 1–13.

YOSHIDA, H., BOGAKI, M., NAKAMURA, M. & NAKAMURA, S. (1990). Quinolone resistance-determining region in the DNA gyrase *gyrA* gene of *Escherichia coli*. *Antimicrob. Agents Chemother.*, **34**, 1271–1272.

YOSHIDA, H., BOGAKI, M., NAKAMURA, M., YAMAKA, L.M. & NAKAMURA, S. (1991). Quinolone resistance-determining region in the DNA gyrase *gyrB* gene of *Escherichia coli*. *Antimicrob. Agents Chemother.*, **35**, 1647–1650.

ZWELLING, L.A., MICHAELS, S., ERICKSON, L.C., UNGERLEIDER, R.S., NICHOLS, M. & KOHN, K.W. (1981). Protein-associated DNA strand breaks in L1210 cells treated with the DNA intercating agents 4′-(9-acridinylamino)methanesulfon-m-anisidide and adriamycin. *Biochemistry*, **20**, 6553–6563.